# Supplementary material for: A hybrid attention network with convolutional neural network and transformer for underwater image restoration
Source: PeerJ Comput Sci. 2023 Nov 7;9:e1559. doi: 10.7717/peerj-cs.1559 (PMC10702994; doi:10.7717/peerj-cs.1559)
Supplement: Supplemental Information 1 [file peerj-cs-09-1559-s001.zip › UWater's code/Readme_Uwater.docx]

**Requirements**

- CUDA 10.1 (or later)
- Python 3.8
- Pytorch 1.8.1
- Torchvision 0.19

A NVIDIA GeForce RTX 3090 GPU

**Prepare Dataset**

- DIV2K/DIV2K_train_HR/x1
- DIV2K/DIV2K_train_LR_bicubic/x1

**Training**

- Run RCAN_TrainCode/code/main_train.py
- Note: args.ext = 'sep' #### Firstly use datasets: **sep_ reset**

**Testing**

- Run RCAN_TestCode/code/main_test.py

**the meaning of the network construction**

RCAN_TrainCode/code/model/rcan.py

def forward(self, x):

x0 = self.sub_mean(x)

####Global feature extraction consists of one Patch embed (3x3 Conv layer), two Transformer Blocks with Self-Attention and and Feed Forward Network (FFN) blocks.

x_p = self.patch_embed(x0) # one Patch embed (3x3 Conv layer).

#### two Transformer Blocks

x_t1 = self.TiB1(x_p)

x_t2 = self.TiB2(x_t1)

#####################

####Local feature extraction includes a Conv layer and 4 residual Channel Attention blocks (RCABs).

x1 = self.head(x0)

x2 = self.body(x1)

x2 = x1 + x2

#####################

#### Feature fusion and enhancement

x3_f, x_sam = self.sam(x2, x)

### Supervised attention(SAM) can generate attention feature maps to suppress features with less information at the moment and only pass useful features to the next stage.

################ Local and global features can be successfully fused to strengthen deep features.

x4 = torch.cat((x_t2, x3_f), 1) # Feature fusion

x5 = self.refinement(x4) #Refinement block includes 4 residual Channel Attention blocks (RCABs) and a 3×3 convolution layer.

###################

x6 = self.tail(x5) # a 3×3 convolution layer to reconstruct image.

x = self.add_mean(x6)

return x, x_sam ###return clear image(x) and attention feature maps(x_sam).
